# Inclusive design in digital mental health: stakeholders with disabilities must be central to usability testing

**DOI:** 10.3389/fdgth.2026.1833693

**Published:** 2026-06-26

**Authors:** Heidi Tranter, Nathan Giles, Thomas Price, Charlotte Stockton-Powdrell, Simon Foster, Sanaullah Alam, Lamiece Hassan, Julian Edbrooke Childs, Marta Chmielowska, Mia Vines Booth, Kathryn M. Abel, Pauline Whelan

**Affiliations:** 1 GM.Digital Research Unit, Research and Innovation, Greater Manchester Mental Health NHS Foundation Trust, Manchester, United Kingdom; 2 Centre for Women’s Mental Health, Division of Psychology & Mental Health, Faculty of Biology, Medicine & Health, University of Manchester, Manchester, United Kingdom; 3 Division of Informatics, Imaging and Data Science, School of Health Sciences, University of Manchester, Manchester, United Kingdom; 4 Centre for Biostatistics, Division of Population Health, Health Services Research and Primary Care, Faculty of Biology Medicine & Health, University of Manchester, Manchester, United Kingdom; 5 Evidence Based Practice Unit, Anna Freud Centre, London, United Kingdom

**Keywords:** accessibility, CAMHS, co-design, digital mental health, inclusive design

## Abstract

Co-developing digital mental health tools is essential to ensure innovations are usable, engaging and accessible for the target populations. The Web Content Accessibility Guidelines, version 2.2, provide the minimum level of compliance for digital mental health tools and these should be further built on through usability testing. Developing digital tools without following inclusive design principles risks widening the digital divide, especially for individuals with disabilities. The Enhancing Child and Adolescent Mental Health Service (CAMHS) Referrals 2 project funded by the National Institute of Health Research developed a digital referral tool for NHS CAMHS. The tool was co-developed with a Pan-Disability Digital Accessibility Group members of which all had lived experience of disabilities. The group advised on the accessibility of the tool through usability testing workshops. As a direct result of the feedback from the workshops, changes were made to the multi-factor authentication and the wording and navigation throughout the tool. This paper outlines the methods and processes followed to develop the group and facilitate the usability testing workshops to provide guidance for future digital mental health research teams. Engagement from advisory groups with lived experience can ensure accessibility is in-built from the start of development and not included as an afterthought.

## 1 Introduction

It is widely acknowledged that digital tools should be clear, usable, engaging and accessible for the populations they are designed to reach (1). The development of digital tools which do not undergo co-design processes risk widening the digital divide for individuals with additional needs (2). The digital divide refers to a broad set of reasons such as age, gender, socioeconomic status, employment status and disability that lead to big differences in how modern technologies can add value to people's lives depending on their ability to access and use them (3). A survey within the Swedish population identified several digital divides affecting those with disabilities compared to the general population (4). These divides include access to devices, disability groups report less access to devices; use of the internet in relation to gender, women with disabilities use the internet more frequently and for more complicated tasks; perception of digital inclusion, some disability groups feel more included in the digital society compared to others, for example those with visual impairments. Embedding inclusive design principles into the software development process can improve usability and accessibility for all users (3, 5, 6).

The National Institute of Health Research (NIHR) Accessibility Commitment (7) states ‘All website and application development work must be compliant with Web Content Accessibility Guidelines (WCAG) version 2.2 to AA standard’ (8). Previous research has called for well-described, contextualized iterative design processes to be documented to create a shared knowledge base for best practice across research teams (9). The NIHR-funded ‘Enhancing CAMHS Referrals 2 (EN-CAMHS 2)’ (NIHR158809) (10) project aims to co-develop a digital referral tool for NHS child and adolescent mental health services (CAMHS) to improve the quality of referrals received and to enhance the referral experience for children and young people. This work follows a previous NIHR-funded project “EN-CAMHS 1” (11) which identified confusion about what CAMHS is for and what it does and does not provide, alongside a lack of support for children and families during the referral process. The EN-CAMHS 2 project aimed to streamline referrals to NHS CAMHS through digital technology enabling better communication with families. This paper outlines how we approached development of the new referral tool to ensure broad accessibility for stakeholders with disabilities; with a specific focus on the usability workshops with the Pan-Disability Digital Accessibility Group (PDDAG).

## 2 Methods

### 2.1 Development of the pan-disability digital accessibility group (PDDAG)

The research team sought advice from an NIHR Expert-by-Experience (Nathan Giles (NG)) to develop the terms of reference and recruit membership to our PDDAG. Initially, three meetings were held between NG and the research team lead (HT) to understand how best the PDDAG would capture a wider perspective of people with disabilities and the criteria for membership were agreed. The EN-CAMHS 2 referral tool has been co-designed for adults and children and young people by the research team and 33 stakeholder participants across 8 focus groups. The ethical approvals in place for this project meant only young people aged 16 or over can use the tool in the context of this research project. Therefore, membership of the PDDAG was open to those aged 16 or over. Ensuring the digital referral tool was accessible for different needs such as neurodivergence and physical disability was identified as a key feature through the “EN-CAMHS 1” focus group discussions. Membership was restricted to a maximum of 15 members, to ensure the team had sufficient time to engage with all members throughout the usability workshops. The total number of members was thus not guided by quantity, but by the concept of ‘information power’ (12), which focuses on quality and comprehensiveness of data. In studies with narrowly defined aims, researchers are encouraged to aim for data sufficiency, not data saturation, which allows for smaller, information-rich samples (13).

### 2.2 Recruitment and sampling

PDDAG members were recruited through opportunity sampling. We prioritized inclusion of applications from people with experience of accessing NHS CAMHS due to the focus of the research project, although this was not an essential. Participants were informed of this during recruitment. NG advised HT to meet with individuals who expressed an interest in joining via Microsoft Teams (14) on a one-to-one basis to explain more about EN-CAMHS 2 and the expectations of the group. These meetings also allowed for HT to understand more about each individual's specific need/s, whether they would prefer to meet online or in person and the preferred reimbursement method (vouchers or bank transfer). Following these meetings 12 members were recruited to the PDDAG for the usability testing workshops which were audio and video recorded (see Table 1 for demographic characteristics). By capturing the self-described primary disability, the research team ensured differing accessibility needs were captured across the group. In addition to the PDDAG (*n* = 12), the EN-CAMHS 2 project has two separate advisory groups made up of unique individuals, one for young people (*n* = 10) and one for parents and professionals (*n* = 10) to provide input throughout the project from non-disabled perspectives, to allow for comparison between groups. The usability workshops outlined in this paper focus solely on the input from the PDDAG members.

**Table 1:** PDDAG demographic characteristics.

Category	Number of PDDG members
Age Range	
16–19	2
20–24	4
25–29	5
30–34	1
Gender	
Male	2
Female	8
Non-binary	2
Ethnicity	
Asian British	1
Mixed (other)	1
Mixed Ethnic Groups - White Latin	1
White British	7
White Scottish	1
Not reported	1
Self-Described Primary Disability (co-morbidities are not included)	
Chronic Pain and Fatigue	4
Physical disability	2
Neurodivergence	4
Visual Impairment	2

### 2.3 Usability workshops

The usability testing workshops were held online and lasted 1–1.5 h. Members were reimbursed for their time at NIHR rates. Members were given the option to split the workshop into two shorter sessions ranging from 30 to 45 min (1/12 members opted for this option; 11/12 joined for the full session). To ensure the workshops were as consistent as possible, a protocol was co-developed by TP, HT and the aforementioned EN-CAMHS 2 young people's advisory group for use during the workshops. Members of the PDDAG were asked to make a referral using the digital referral tool with limited input from a member of the research team. The first round of workshops were held in July 2025 (*n* = 12); feedback was then reviewed by the Research Associate (TP), HT and Co-Principal Investigator (CSP) and Digital Health Software team at the University of Manchester to determine adaptations which needed to be made to the tool. A second round of one-to-one usability testing workshops were held in September 2025 (*n* = 12) with the same members of the PDDAG to review the changes which had been made. The second round of testing allowed members of the PDDAG to review alterations to the tool and observe the impact of their involvement. This served to ensure tool improvement and provide assurance to members of the PDDAG that their engagement was meaningful.

## 3 Results

The feedback from the PDDAG on the digital referral tool was overwhelmingly positive. The group found the tool understandable and convenient. Similar to the approach adopted by Whelan et al (6). This project involved User-design in the Alpha (second) phase of the three UK Government Service Manual, Discovery, Alpha and Beta (15). The Alpha phase involved the following stages:

1. Members of the PDDAG completed a workshop with a member of the project team to make a referral to CAMHS using the digital tool.
2. The technical team adapted the tool based on the feedback from the first workshop.
3. Members of the PDDAG attended a second workshop with a member of the project team to ensure the adaptations made by the technical team reflected their feedback.

TP collated notes made by members of the research team during the workshop, and listened back to the audio and video recordings of each workshop to make sure all of the feedback was captured. Feedback was then collated into themes to identify features which either needed to be included or adapted by the technical team. These were discussed and agreed with the Co-Principal Investigator (CSP) and Project Manager (HT). The project team then met with the technical team to prioritize the adaptations to the tool using the MoSCoW framework, aligned with Agile approaches (16). This approach is often used in software development to prioritize features/deliverables across mixed stakeholder discussions. The MoSCoW approach saw the teams rank adaptations as ‘must have’, ’should have’ ‘could have’ or won't have’. ‘Must have’ adaptations were seen as essential by stakeholders; ’should have’ adaptations, whilst seen as important, would not affect the functionality of the tool if they were not implemented; ‘could have’ adaptations were additional features, which were not necessarily related to the aims of the EN-CAMHS 2 project; and adaptations deemed as ‘won't have’ were either not priorities for stakeholders and/or not deliverable within the scope of the study. The main themes from the workshops are shown in Table 2, these are (1) multi-factor authentication (MFA); (2) specific updates to form sections; (3) navigation; and (4) positive feedback. Each of the feedback items were considered in a follow up meeting with the research team and technical team to determine if changes needed to be made to the tool, and if so, what these were. We aimed to implement as many of the feedback items as possible, within the time and budget of the research project. Table 2 includes a ‘Project Solution’ column which details the changes made to the referral tool in response to each feedback item.

**Table 2:** Usability testing workshop feedback items and project solutions.

Feedback item	Project solution
1. Multi-Factor Authentication (MFA)	
Unclear as to how to scan the QR code for MFA. Members did this using the camera when they needed to download the Microsoft Authenticator app and scan the QR code through this.	Development and inclusion of step-by-step guide to complete the MFA process.
Buttons were unclear, members did not know where to click.	Changed buttons from grey text to large white text on blue button (consistent with the rest of the form).
If this section of the form could not be completed, then the user would not be able to progress with form. This could be a specific problem for people who only have one device, as setting up MFA requires two devices.	Included an alternative option to complete MFA, by email, as opposed to just scanning a QR code.
Screen reader did not pick up the QR code image or the section to enter the code from the authenticator app.	Added alt text which describes what the image is for and where to enter the code for people who have visual impairments.
2. Specific updates to form sections	
‘Your Details’ section of the form	
Changing ‘alias’ as it is not clear what this term means.	‘Alias’ has been updated to ‘any other names’.
Repetition of questions relating to language and communication needs	Updated the form so this question is only included once.
Unclear as to what Child Protection Plan means, when ‘Child in Need’ has an information button to explain this.	Added an information button to explain ‘Child Protection Plan’.
‘Responsible Adult’ section of the form	
Large amount of text on one page, which felt overwhelming and confusing.	Separated this out across two pages of the form.
‘Additional Details’ section of the form	
Question about parents/carers mental health is repeated twice.	Updated the form so this question is only included once.
‘Consent’ section of the form	
There should be a minimum level of consent required, if someone does not consent for the referral to be shared with CAMHS then the form should not be sent.	Updated so if the person completing the form selects ‘no’ to this question, the form is not sent to CAMHS and this is explained to the person filling out the form.
Too many consent questions on one page.	Separated the consent questions out across three pages (one per page).
3. Navigation	
Include back buttons at the end of the form to allow navigation back to different sections, without having to go back through the whole form.	This has been updated to it is easier to navigate back to specific sections of the form to update these before submitting.
Inclusion of a progress bar to visually show how much of the form has been completed and how much is left.	This has been included.
4. Positive Feedback	
Good use of drop-down explanations throughout the tool.	No solution from project team needed.
Navigation through the questions, streamlined.	
The standard NHS template, it looked nice and was easy to read.	
Good flow of questions.	
The form felt short, less repetitive and to the point.	
Liked the option to download a PDF version of the completed form.	
Person-friendly, less system driven.	
Positive user experience.	
Good use of prompts in the free-text questions.	
This form seems much clearer than previous referral forms.	
More streamlined – clearer what to do on each section, better prompts/formatting.	
‘…changed in the right direction…’	

The EN-CAMHS 2 referral form is split into multiple sections, to avoid overwhelming referrers with a lengthy series of questions. Feedback from previous co-design work indicated the inclusion of how long it would take a referrer to complete each section of the referral form i.e., an estimated completion time would be useful. During the workshops, the research team timed how long each member of the PDDAG took to complete each section of the referral form so accurate estimated times to complete could be provided. The estimated completion times for each section of the form were tested during the workshops. Stakeholders outside the PDDAG had assumed that estimated completion times for disabled people were an underestimate and would be longer. The workshops found the estimated completion times for each section were accurate for able and disabled groups. Completion times for each section of the form by the PDDAG are shown in Table 3. We found no difference in completion times for the able and disabled groups.

**Table 3:** Completion times for each section of the digital referral form.

Digital referral form section	Completion time (minutes)
Child or Young Person's Details	5
Child or Young Person's GP Details	5
Child or Young Person's School or College Details	5
Other services working with the Child or Young Person	3
Referral Details	15
Child or Young Person's Needs	5
Consent	5

Note there were no differences in completion times for the PDDAG and other project advisory groups.

## 4 Discussion

During the co-development of the EN-CAMHS 2 digital referral tool with PDDAG stakeholders, the main findings were that the tool followed a clear process for making a referral to CAMHS and the content was clear and easy to understand. The feedback from the PDDAG identified improvements while achieving a balance between repeated concerns and novel experiences. The main improvements made to the tool as part of the usability workshops were the wording and options for MFA and simplifying the navigation throughout the tool. Specific updates in terms of wording, providing additional information and spreading out content over multiple pages were made to certain sections within the form. The feedback allowed the research team and technical team to incorporate changes which are likely to have a positive influence on the usability of the tool for future users. Updates to the EN-CAMHS 2 referral tool based on PDDAG feedback were reviewed by the other project advisory groups to ensure accessibility from non-disabled perspectives.

The value of the PDDAG in creating a better tool for all users is verified by the increase of positive approvals made during the second round of testing with all users benefitting from alterations not just those who required the adaptation. Following PDDAG feedback, we made changes aiming to improve the visual appeal of the tool and reduce the level of digital competency and essential skills required. This parallels a finding of Kim et al. (17) in which application of accessibility focused user experience guidelines improved information recognition and uptake of a digital tool for both those with a visual impairment and for those without. Adopting an inclusive design process is consistent with previous research (3) that usability and accessibility for all users were improved as a result. As EN-CAMHS 2 looks to develop a national referral tool for children and young people, the PDDAG included stakeholders to represent a wide spectrum of disabilities. This meant feedback was more general and broader, as opposed to being representative of specific disabilities and diagnoses.

Whilst the research team made every effort to include a diversity of needs and backgrounds, representative of the accessibility needs identified in “EN-CAMHS 1”, one of the challenges with this may be the feedback was oversimplified, therefore the tool may not be wholly adapted for specific needs. The ethical approval for this project was for participants aged 16 or over, therefore future accessibility work should adopt structured, purposive sampling to adapt the tool for use with children and young people under 16 years of age. The digital referral tool has been designed for use in NHS CAMHS, some of these services accept self-referrals for children aged under 16, therefore the tool would need to be accessible for younger children. We were unable to recruit individuals with a hearing impairment during the time frame; as a result of this we are unsure as to how accessible the digital referral tool is for people whose first language is British Sign Language (BSL). The digital referral tool is not compatible with BSL interaction as we lacked time, resource and access to expertise. We worked with the Manchester Deaf Centre charity to develop a BSL video which is available on the homepage to explain the tool has not been translated into BSL and advise the referrer to follow local guidance to access CAMHS support. Future work should seek to include those with BSL as a first language in the early development stage of any digital technology, exploring the difficulties with interaction-based tools and reimagining how this can be achieved.

Although technology development now realizes the need of end user input, that input often overlooks the distinct needs of disabled people who are likely also to want to use it. Much of the literature addresses the role of adapted technology integrating those with disabilities; rather than embedding accessibility within the technology development (18, 19). These authors emphasize the importance of engaging multiple stakeholders, particularly disabled individuals early in the creation and adoption of digital technologies. Co-creation ensures digital health interventions are user-centered and meet the specific needs of target populations, and is pivotal for enhancing outcomes for digital public health interventions (20).

Adopting a co-development approach in research with relevant stakeholder groups and individuals who have additional needs or are registered as disabled, incorporates feedback directly into the design and development of digital tools (21). The triangulation of different stakeholder viewpoints enables research teams to design tools which are truly as accessible as possible (8). Collaborative co-design with lived experience following an iterative process should be included within the research design of digital tools to empower the voices of stakeholders (1). Following an iterative, agile design process allows for the diverse range of accessibility needs to be incorporated as best as possible.

To the best of our knowledge, this is one of few studies which has specifically involved a PDDAG to advise on the research process with a specific focus on digital technology. Our experience of developing and involving the PDDAG is consistent with previous research which emphasizes the need for inclusivity and research teams being reflexive and iterative in how they work with disability advisory groups (22). By following this approach, we ensured stakeholders felt comfortable in contributing to the research project, enabling us to incorporate their feedback which is crucial as feeling valued is a key factor in maintaining Patient and Public Involvement PPI activity (23). Our PDDAG was also approached by other projects (24) and digital tools (25) to ensure their accessibility for disabled people, following similar testing procedures.

## 5 Conclusion

Creation and involvement of the PDDAG in the EN-CAMHS 2 project led to several accessibility enhancing changes to the presentation of the technology which are likely to have a positive influence on successfully embedding the tool in the future. The usability testing process reiterated how a tool such as this could enhance access to appropriate care, especially for neurodivergent and disabled individuals. Previous research called for better documentation of well-described, contextualized iterative design processes to create a shared knowledge base for best practice across research teams. We consider that developing digital health tools in line with the WGAG 2.2 should be a minimum requirement (for accessibility) and that usability testing should become routine with advisory groups such as our PDDAG. Such engagement from advisory groups with lived experience of disability is far more likely to ensure accessibility is in-built. Future research should focus on the needs of people with disability which can be better reflected in treatment and support provided through digital innovations.

## Data Availability

The original contributions presented in the study are included in the article/Supplementary Material, further inquiries can be directed to the corresponding author.
